# Supplementary material for: A UMLS-based spell checker for natural language processing in vaccine safety
Source: BMC Med Inform Decis Mak. 2007 Feb 12;7:3. doi: 10.1186/1472-6947-7-3 (PMC1805499; doi:10.1186/1472-6947-7-3)
Supplement: Additional file 13 — RAP application directory. Sets up directory for RAP (RDF application for PHP) source code [file 1472-6947-7-3-S13.gz › rap/doc/phpdoc/resModel/ResModel.html]

xml version="1.0" encoding="iso-8859-1"?


Docs For Class ResModel


## Class ResModel

Description

Description |
Descendents
| Methods (details)

A ResModel provides an resource centric view on an underlying RDF model.

ResModels show information not as statements but as resources with properties, similar to Jena models. ResModels may create Resources [URI nodes and bnodes]. Creating a Resource does not make the Resource visible to the model; Resources are only "in" Models if Statements about them are added to the Model. Similarly the only way to "remove" a Resource from a Model is to remove all the Statements that mention it.

When a Resource or Literal is created by a Model, the Model is free to re-use an existing Resource or Literal object with the correct values, or it may create a fresh one.

History: <LI>09-10-2004 : First version of this class.</LI>

- access: public
- author: Daniel Westphal <at d-westphal dot de mail at d-westphal dot de>
- version: V0.9.1

Located in /resModel/ResModel.php (line 29)

Direct descendents

Description |
Descendents
| Methods (details)

| Class | Description |
| --- | --- |
| OntModel | Enhanced view of the model that is known to contain ontology data, under a |

Method Summary

Description |
Descendents |
Methods (details)

ResModel
ResModel
(object model &$model)

void
add
(object Statement $statement)

void
addModel
(object Model &$model)

void
addNamespace
(mixed $prefix, mixed $namespace, String $prefix,)

void
addParsedNamespaces
(Array $newNs)

boolean
addWithoutDuplicates
(object Statement $statement)

boolean
contains
(object Statement &$statement)

boolean
containsAll
(object Model &$model)

boolean
containsAny
(object Model &$model)

boolean
containsResource
(object Node &$node)

object ResProperty
createAlt
([string $uri = null])

object ResProperty
createBag
([string $uri = null])

object ResProperty
createList
([string $uri = null])

object ResLiteral
createLiteral
(string $label, [string $languageTag = null])

object ResProperty
createProperty
([string $uri = null])

object ResResource
createResource
([string $uri = null])

object ResProperty
createSeq
([string $uri = null])

object ResLiteral
createTypedLiteral
(string $value, string $dtype)

boolean
equals
(object model &$that)

array
find
(object ResResource $subject, object ResResource $predicate, object ResResource $object)

object Statement
findFirstMatchingStatement
(object Node $subject, object Node $predicate, object Node $object, integer $offset)

string
getBaseURI
()

object Model
&getModel
()

Array
getParsedNamespaces
()

object Statement
getProperty
(object ResResource $subject, object ResResource $property)

object MemModel
&intersect
(object Model &$model)

boolean
isEmpty
()

object ResIterator
listObjects
()

object ResIterator
listObjectsOfProperty
(object ResResource $property, [object ResResource $value = null])

object ResIterator
listSubjects
()

object ResIterator
listSubjectsWithProperty
(object ResResource $property, [object ResResource $value = null])

void
load
(string $filename, [string $type = NULL], [boolean $stream = false])

array
&rdqlQuery
(string $queryString, [boolean $returnNodes = TRUE])

object RdqlResultIterator
rdqlQueryAsIterator
(string $queryString, [boolean $returnNodes = TRUE])

boolean
remove
(object Statement $statement)

void
removeNamespace
(String $nmsp)

boolean
saveAs
(string $filename, [string $type = 'rdf'])

void
setBaseURI
(string $uri)

integer
size
()

object MemModel
subtract
(object Model $model)

object MemModel
&unite
(object Model &$model)

void
writeAsHTMLTable
()

string
writeRdfToString
()

Methods

Description |
Descendents |
Methods (details)

Constructor ResModel (line 47)

Constructor You have to supply a memmodel/dbmodel/infmodel to save the statements.

- access: public

ResModel

ResModel
(object model &$model)

- object model
  $model

add (line 199)

Adds a new triple to the Model without checking if the statement is already in the Model.

So if you want a duplicate free Model use the addWithoutDuplicates() function (which is slower then add()) Expects a statements with ResResources(ResLiterals)

- throws: PhpError
- access: public

void

add
(object Statement $statement)

- object Statement
  $statement

addModel (line 510)

Adds another model to this MemModel.

Duplicate statements are not removed. If you don't want duplicates, use unite(). If any statement of the model to be added to this model contains a blankNode with an identifier already existing in this model, a new blankNode is generated.

- throws: phpErrpr
- access: public

void

addModel
(object Model &$model)

- object Model
  $model

addNamespace (line 855)

Adds a namespace and prefix to the model.

- access: public
- author: Tobias Gau� <tobias.gauss@web.de>

void

addNamespace
(mixed $prefix, mixed $namespace, String $prefix,)

- String
  $prefix,: String $nmsp

addParsedNamespaces (line 843)

Adds the namespaces to the model. This method is called by the parser. !!!! addParsedNamespaces() not overwrites manual added namespaces in the model !!!!

- access: public
- author: Tobias Gau� <tobias.gauss@web.de>

void

addParsedNamespaces
(Array $newNs)

- Array
  $newNs

addWithoutDuplicates (line 218)

Checks if a new statement is already in the Model and adds the statement, if it is not in the Model.

addWithoutDuplicates() is significantly slower then add(). Retruns TRUE if the statement is added. FALSE otherwise. Expects a statements with ResResources(ResLiterals)

- throws: PhpError
- access: public

boolean

addWithoutDuplicates
(object Statement $statement)

- object Statement
  $statement

contains (line 236)

Tests if the Model contains the given statement.

TRUE if the statement belongs to the model; FALSE otherwise. Expects a statement of ResResources(ResLiterals)

- access: public

boolean

contains
(object Statement &$statement)

- object Statement
  $statement

containsAll (line 253)

Determine if all of the statements in a model are also contained in this model.

True if all of the statements in $model are also contained in this model and false otherwise.

- access: public

boolean

containsAll
(object Model &$model)

- object Model
  &$model

containsAny (line 269)

Determine if any of the statements in a model are also contained in this model.

True if any of the statements in $model are also contained in this model and false otherwise.

- access: public

boolean

containsAny
(object Model &$model)

- object Model
  &$model

containsResource (line 283)

Determine if the node (ResResource / ResLiteral) $node appears in any statement of this model.

- access: public

boolean

containsResource
(object Node &$node)

- object Node
  &$node

createAlt (line 529)

Create a new RDF Container from type rdf:Alt This method may return an existing container with the correct URI and model, or it may construct a fresh one, as it sees fit.

Subsequent operations on the returned Container may modify this model.

- access: public

object ResProperty

createAlt
([string $uri = null])

- string
  $uri

createBag (line 549)

Create a new RDF Container from type rdf:Bag This method may return an existing container with the correct URI and model, or it may construct a fresh one, as it sees fit.

Subsequent operations on the returned Container may modify this model.

- access: public

object ResProperty

createBag
([string $uri = null])

- string
  $uri

createList (line 589)

Create a new RDF Collection from type rdf:List This method may return an existing container with the correct URI and model, or it may construct a fresh one, as it sees fit.

Subsequent operations on the returned Container may modify this model.

- access: public

object ResProperty

createList
([string $uri = null])

- string
  $uri

createLiteral (line 113)

Create an untyped literal from a String value with a specified language.

If you want to type this literal, you have to set a datatype before adding it to the model.

- access: public

object ResLiteral

createLiteral
(string $label, [string $languageTag = null])

- string
  $label
- string
  $languageTag

createProperty (line 93)

Create a new Property associated with this model.

This method may return an existing property with the correct URI and model, or it may construct a fresh one, as it sees fit.

Subsequent operations on the returned property may modify this model.

- access: public

object ResProperty

createProperty
([string $uri = null])

- string
  $uri

createResource (line 71)

Create a new resource associated with this model.

If the uri string isn't set, this creates a bnode. Otherwise it creates a URI node. A URI resource is .equals() to any other URI Resource with the same URI (even in a different model - be warned).

This method may return an existing Resource with the correct URI and model, or it may construct a fresh one, as it sees fit.

Operations on the result Resource may change this model.

- access: public

object ResResource

createResource
([string $uri = null])

- string
  $uri

createSeq (line 569)

Create a new RDF Container from type rdf:Seq This method may return an existing container with the correct URI and model, or it may construct a fresh one, as it sees fit.

Subsequent operations on the returned Container may modify this model.

- access: public

object ResProperty

createSeq
([string $uri = null])

- string
  $uri

createTypedLiteral (line 302)

Create a literal from a String value with the $dtype Datatype An existing literal of the right value may be returned, or a fresh one created.

- access: public

object ResLiteral

createTypedLiteral
(string $value, string $dtype)

- string
  $value
- string
  $dtype

equals (line 323)

Checks if two models are equal.

Two models are equal if and only if the two RDF graphs they represent are isomorphic.

Warning: This method doesn't work correct with models where the same blank node has different identifiers in the two models. We will correct this in a future version.

- throws: phpErrpr
- access: public

boolean

equals
(object model &$that)

- object model
  &$that

find (line 135)

General method to search for triples.

NULL input for any parameter will match anything. Example: $result = $m->find( NULL, NULL, $node ); Finds all Statements with $node as object. Returns an array of statements with ResResources.

- throws: PhpError
- access: public

array

find
(object ResResource $subject, object ResResource $predicate, object ResResource $object)

- object ResResource
  $subject
- object ResResource
  $predicate
- object ResResource
  $object

findFirstMatchingStatement (line 170)

Searches for triples and returns the first matching statement.

NULL input for any parameter will match anything. Example: $result = $m->findFirstMatchingStatement( NULL, NULL, $node ); Returns the first statement with ResResources of the Model where the object equals $node. Returns an NULL if nothing is found. You can define an offset to search.

- access: public

object Statement

findFirstMatchingStatement
(object Node $subject, object Node $predicate, object Node $object, integer $offset)

- object Node
  $subject
- object Node
  $predicate
- object Node
  $object
- integer
  $offset

getBaseURI (line 646)

Return current baseURI.

- access: public

string

getBaseURI
()

getModel (line 604)

Returns a reference to the underlying model (Mem/DB/InfModel) that contains the statements

- access: public

object Model

&getModel
()

getParsedNamespaces (line 828)

Returns the models namespaces.

- access: public
- author: Tobias Gau� <tobias.gauss@web.de>

Array

getParsedNamespaces
()

getProperty (line 355)

Answer a statement find(s, p, null) with ResResources(ResLiterals) from this model.

If none exist, return null; if several exist, pick one arbitrarily.

- throws: phpErrpr
- access: public

object Statement

getProperty
(object ResResource $subject, object ResResource $property)

- object ResResource
  $subject
- object ResResource
  $property

intersect (line 687)

Returns a new model containing all the statements which are in both this model and another.

- throws: phpErrpr
- access: public

object MemModel

&intersect
(object Model &$model)

- object Model
  $model

isEmpty (line 378)

Checks if MemModel is empty

- access: public

boolean

isEmpty
()

listObjects (line 390)

Returns a ResIterator with all objects in a model.

- throws: phpErrpr
- access: public

object ResIterator

listObjects
()

listObjectsOfProperty (line 403)

Returns a ResIterator with all objects with a given property and property value.

- access: public

object ResIterator

listObjectsOfProperty
(object ResResource $property, [object ResResource $value = null])

- object ResResource
  $property
- object ResResource
  $value

listSubjects (line 416)

Returns a ResIterator with all subjects in a model.

- throws: phpErrpr
- access: public

object ResIterator

listSubjects
()

listSubjectsWithProperty (line 430)

Returns a ResIterator with all subjects with a given property and property value.

- throws: phpErrpr
- access: public

object ResIterator

listSubjectsWithProperty
(object ResResource $property, [object ResResource $value = null])

- object ResResource
  $property
- object ResResource
  $value

load (line 635)

Load a model from a file containing RDF, N3 or N-Triples.

This function recognizes the suffix of the filename (.n3 or .rdf) and calls a suitable parser, if no $type is given as string ("rdf" "n3" "nt"); If the model is not empty, the contents of the file is added to this DbModel.

- access: public

void

load
(string $filename, [string $type = NULL], [boolean $stream = false])

- string
  $filename
- string
  $type
- boolean
  $stream

rdqlQuery (line 797)

Perform an RDQL query on this MemModel.

This method returns an associative array of variable bindings. The values of the query variables can either be RAP's objects (instances of Node) if $returnNodes set to TRUE, or their string serialization.

- return: [][?VARNAME] = object Node (if $returnNodes = TRUE) OR array [][?VARNAME] = string
- access: public

array

&rdqlQuery
(string $queryString, [boolean $returnNodes = TRUE])

- string
  $queryString
- boolean
  $returnNodes

rdqlQueryAsIterator (line 815)

Perform an RDQL query on this MemModel.

This method returns an RdqlResultIterator of variable bindings. The values of the query variables can either be RAP's objects (instances of Node) if $returnNodes set to TRUE, or their string serialization.

- return: = with values as object Node (if $returnNodes = TRUE) OR object RdqlResultIterator = with values as strings if (if $returnNodes = FALSE)
- access: public

object RdqlResultIterator

rdqlQueryAsIterator
(string $queryString, [boolean $returnNodes = TRUE])

- string
  $queryString
- boolean
  $returnNodes

remove (line 445)

Removes the statement of ResResources(ResTriples) from the MemModel.

TRUE if the statement is removed. FALSE otherwise.

- throws: PhpError
- access: public

boolean

remove
(object Statement $statement)

- object Statement
  $statement

removeNamespace (line 866)

removes a single namespace from the model

- access: public
- author: Tobias Gau� <tobias.gauss@web.de>

void

removeNamespace
(String $nmsp)

- String
  $nmsp

saveAs (line 664)

Saves the RDF,N3 or N-Triple serialization of the MemModel to a file.

You can decide to which format the model should be serialized by using a corresponding suffix-string as $type parameter. If no $type parameter is placed this method will serialize the model to XML/RDF format. Returns FALSE if the MemModel couldn't be saved to the file.

- access: public

boolean

saveAs
(string $filename, [string $type = 'rdf'])

- string
  $filename
- string
  $type

setBaseURI (line 768)

Set a base URI for the MemModel.

Affects creating of new resources and serialization syntax. If the URI doesn't end with # : or /, then a # is added to the URI.

- access: public

void

setBaseURI
(string $uri)

- string
  $uri

size (line 459)

Number of statements in the MemModel

- access: public

integer

size
()

subtract (line 338)

Returns a new model that is the subtraction of another model from this model.

- throws: phpErrpr
- access: public

object MemModel

subtract
(object Model $model)

- object Model
  $model

unite (line 491)

Returns a new Model that is the set-union of the model with another model.

Duplicate statements are removed. If you want to allow duplicates, use addModel() which is much faster.

The result of taking the set-union of two or more RDF graphs (i.e. sets of triples) is another graph, which we will call the merge of the graphs. Each of the original graphs is a subgraph of the merged graph. Notice that when forming a merged graph, two occurrences of a given uriref or literal as nodes in two different graphs become a single node in the union graph (since by definition they are the same uriref or literal) but blank nodes are not 'merged' in this way; and arcs are of course never merged. In particular, this means that every blank node in a merged graph can be identified as coming from one particular graph in the original set of graphs.

Notice that one does not, in general, obtain the merge of a set of graphs by concatenating their corresponding N-triples documents and constructing the graph described by the merged document, since if some of the documents use the same node identifiers, the merged document will describe a graph in which some of the blank nodes have been 'accidentally' merged. To merge Ntriples documents it is necessary to check if the same nodeID is used in two or more documents, and to replace it with a distinct nodeID in each of them, before merging the documents. (Not implemented yet !!!!!!!!!!!)

- throws: phpErrpr
- access: public

object MemModel

&unite
(object Model &$model)

- object Model
  $model

writeAsHTMLTable (line 674)

Writes the RDF serialization of the MemModel as HTML table.

- access: public

void

writeAsHTMLTable
()

writeRdfToString (line 779)

Writes the RDF serialization of the MemModel as HTML table.

- access: public

string

writeRdfToString
()

Documentation generated on Thu, 7 Jul 2005 13:43:43 +0200 by phpDocumentor 1.3.0RC3
